# Vaya Con Dios: The Influence of Religious Constructs on Stressors around the Migration Process and U.S. Lived Experiences among Latina/o Immigrants

**DOI:** 10.3390/ijerph17113961

**Published:** 2020-06-03

**Authors:** Oswaldo Moreno, Miriam Ortiz, Lisa Fuentes, Dina Garcia, Gabriela Leon-Perez

**Affiliations:** 1 Psychology Department, Virginia Commonwealth University (VCU), Richmond, VA 23284, USA; fuentesls@mymail.vcu.edu; 2 VCU School of Medicine Center on Health Disparities, Richmond, VA 23284, USA; ortizm@mymail.vcu.edu; 3 VCU Department of Health Behavior and Policy, Richmond, VA 23284, USA; dina.garcia@vcuhealth.org; 4 VCU Department of Sociology, Richmond, VA 23284, USA; gleonperez@vcu.edu

**Keywords:** religious practices, Latina/o, immigration, lived experiences, cultural stressors

## Abstract

This qualitative study explores the role of religious practices on the migration process and the U.S. lived experiences of Latina/o immigrants. We conducted semi-structured interviews with 20 Latino/a immigrant adults living in a southern state of the United States. Interviews focused on participants’ migration experiences, religious constructs, and stress responses. Results revealed that religious practices provided strength, well-being, and positive life outlook during the migration process. After migration, religious practices also assisted participants in creating a sense of community/family, as well as provided financial and social support during difficult times. Recommendations for future interdisciplinary research and for practitioners are discussed for individuals working with Latinx and immigrant populations.

## 1. Introduction

The Latina/o population is the largest ethnic minority group in the United States (U.S.) [1]. There are a variety of reasons why Latina/o individuals decide to migrate to the U.S. Although Torres and Wallace [2] note that these reasons vary by country of origin, Latina/o immigrants typically migrate due to lack of financial resources, family obligations, as well as social-political factors encountered in their country of origin [3]. While migrating to the U.S. may be perceived as a positive outlook towards one’s life due to the possibility of gaining resources [4], those who migrate, especially those without legal status, may face additional lost resources including loss of safety stemming from fear of deportation [5,6]. Fear of deportation, even for individuals who have a legal status in the U.S., not only instills stress, but also creates distrust towards public establishments such as legal and social services [7,8,9,10]. Additionally, despite moving to the U.S. to gain financial prosperity [4,11], Latina/o immigrants continue to report financial and work-related stressors while living in the U.S. [2,12]. Even though recent literature provides consistent findings as it pertains to the U.S. lived experiences of Latina/o immigrants, little is known on how religious practice shapes these experiences, especially when resources (e.g., familial and financial support resources) are lost. More research is needed to investigate the role of religious practice among Latina/o immigrants, especially given that over 80% of Latina/o immigrants identify as highly religious [13]. This qualitative paper will therefore explore the role of religious practice on the migration process and U.S. living experiences of foreign-born Latina/os.

### 1.1. Religious Practice: A Conceptualization

Religious practice has been defined as a multidimensional construct [14,15] and involves having similar belief systems and moral values about a divine or a higher power [14]. Religious practices often encompass behavioral patterns, rituals, organizational practices, commitment and social relations that are adherent to an organized religious institution [14]. Many who engage in religious practices also engage in spiritual practices, defined as practices and approaches to life and ties to other people [16], as well as a journey towards a sense of purpose [14,17,18]. Spirituality therefore may serve as a form of personal connection towards a higher power and may not necessarily include being part of a religious institution [14]. For the purpose of this study, however, we define religious practices as engaging in certain practices and rituals through an organized system of beliefs acknowledging the possible overlap between religiosity and spirituality among some Latina/o individuals.

### 1.2. Religious Practices and the Migration Process

Religious practices play a central role in the lives of individuals especially when facing adversity and stress [19,20]. For Latina/o immigrants, challenges and adversity may arise during the migration process to the U.S. [21]. For example, the migration process may be a period of lost resources for undocumented Latina/o immigrants (i.e., material and social resources), which may consequently impact their physical and psychological health. Given that studies have shown that religious practices serve as coping responses among Latinas/os when dealing with difficult life events [20,22], further exploration of the role of religiosity during the migration process is warranted.

### 1.3. Religious Practices and Lived U.S. Experiences

Upon arriving to the U.S., Latina/o immigrants encounter a number of stressors—many of which may be compacted with acculturative stress [23], idioms of distress [24,25], and immigration stressors [4,23,25,26]. On top of the daily stressors faced in the U.S., heightened stress may also be connected to legal status [11], fear of deportation [27,28] and heightened ethnic discrimination [23,24,29,30,31].

The overarching psychology of religion literature suggests that religious support via faith-based institutions provides individuals with social support, meaning making, and perceived life satisfaction when confronted with stressors [32,33]. Given that religion plays a proactive role in the lives of individuals, religious support via faith-based institutions may also provide Latina/o immigrants with a secure environment to maintain their social and ethnic bonds, and to connect with people who have similar cultural traditions and beliefs [32]. Therefore, due to the role that religiosity plays in the lives of Latinas/os, it may also assist Latina/o immigrants to cope with stressors directly related to their lived experience in the U.S. [34].

Empirical research has supported such theories. For example, studies have consistently found that religious practices can function as a social support network to cope with life stressors of Latinas/os in the U.S. [24,32,35,36,37]. However, there are mixed findings about religiosity, stress, and perceived well-being when comparing the Latina/o experience pre- and post-migration. For example, Sanchez and colleagues [22], using a sample of 527 recent immigrants, found that Latinas/os who were religious in their country of origin, experience higher levels of acculturative stress due to the loss of social support experienced upon immigrating to the U.S. On the other hand, Steffen and Merril [37], using a sample of 336 Mexican immigrants, found that pre-immigration religiosity and attending the same religious organization once they immigrate to the U.S. helps Latinas/os interact positively with members of the mainstream culture in the U.S. Other studies provide empirical evidence supporting the theory that religious practices serve as a protective factor for psychological and physical health [36,38]. However, little is still known about the direct role of religious practices on the migration process and U.S. living experiences. Additionally, given that many religious systems (e.g., churches) endorse religious values and morals that impact how Latinas/os approach and experience stressors, as well as their willingness to seek support [39], more research should explicitly examine these direct associations.

### 1.4. Theoretical Framework: Conservation of Resources Theory

Conservation of resources theory (CORT) suggests that individuals aim to attain, preserve, shield, and nurture valued resources, and that stress emerges when these resources are lost [40]. More specifically, Hobfoll [40] argues that psychological stress may arise when individuals face a threat of lost resources and when an investment of resources does not yield the expected return. Hobfoll [40] also places greater emphasis on the shared versus personal nature of stress appraisals suggesting that these lost resources, in most cases, may also be lost with others in similar circumstances. Given that migration is a process that may derive from a lack of resources, CORT may assist in describing stressors during the migration process of Latina/o individuals, as well as understanding how religiosity may play a role during these stressors. For example, the decision to migrate is often marked by major resource loss or conflict through socio-political factors [41], financial stress [4,28] and/or family reunification [42]. The migration process may also be a period of lost resources (e.g., physical and psychological health, material and social resources; [43]) that may continue while living in the new social cultural environment (like the U.S.; [27,44]). For example, the limited resources possessed in their native country may be lost in the new country, thus producing additional stress. CORT may assist in understanding post-migration religious experience. That is, religious practices may play a key role in stress reduction during the migration process and U.S.-lived experience.

### 1.5. Current Study

Through a CORT framework, Latina/o immigrants are a vulnerable population that are exposed to several migration-related stressors and lost resources while migrating and living in the U.S. [27]. These stressors are detrimental to their physical and mental health [4]. Most striking, given that Latina/o immigrants are less likely to seek social and health-related services when compared to their U.S. born counterparts [41,45], this population may seek alternative ways to handle these stressors, such as religious-oriented behaviors. It is estimated that over 80% of first-generation Latina/os identify as highly religious [13]. As such, more research is needed to investigate whether and how religious practices help Latina/o migrants cope with immigration-related stressors. This qualitative paper will therefore explore the role of religious practices on the migration process and U.S. lived experiences. Specifically, this study will explore two overarching research questions: (1) What role do religious practices play on the migration process of Latina/o immigrants? (2) What role do religious practices play on the U.S. living experiences of Latina/o immigrants?

## 2. Methods

### 2.1. Design and Participants

A semi-structured interview was used to elicit responses on migration, religious constructs, methods of coping and stress responses. Given the sensitive nature of the political climate in the U.S., participants did not report their current immigration status (e.g., visas, permanent resident status or U.S. citizenship), although the majority described their migration process through stressful means (therefore following the CORT framework). Of these twenty participants, nine were male (45%) and 11 were female (55%), five (25%) reported speaking English fluently, fifteen (75%) reported either being married or cohabitating and eighteen (90%) reported being religiously committed and adhering to religious practices in a given week. Participants’ average age was 41.0 (SD = 12.20).

### 2.2. Procedures

Inclusion criteria included individuals who self-identified as Latina/o immigrants, were 18 years of age, spoke Spanish or English, did not have any cognitive disorders, and were willing to describe their migration process. Data was collected in a southern state of the United States using community-based participatory research. Specifically, participants were recruited from Latina/o community-based center, clinics, barber shops, etc. Participants were first approached to introduce the study using a brief protocol. If participants expressed interest in the study, consent was then provided. After participants consented, participants then underwent the semi-structured interview in a private and confidential space. Interviews, which lasted about an hour long, were conducted in either Spanish or English and were audio-recorded. All participants were compensated with U.S. $20. This study was approved by the university institutional review board.

### 2.3. Material

The interview guide covered topics including descriptions of religious practices, migration, U.S. lived experiences, descriptions of well-being, as well as connecting experiences and stressors/problems. The present study primarily draws from responses to four interview questions related to religiosity:

1. What kind of religious practices do you engage in during your typical week?
2. How have your religious practices played a role in the immigration process?
3. How did your religious practices help you while you lived here in America?
4. How has it been living in the United States as a first-generation Mexican immigrant?

### 2.4. Data Analytic Plan

While participants were not asked about their legal status, many of them described entering the country without having a visa or avoiding ports of entry (i.e., crossing a desert, river, etc.). These individuals were classified as undocumented at the time of migration.

Interviews were transcribed verbatim in the language in which they were conducted (16 were in Spanish and 6 were in English). Transcripts were coded by three bilingual Latina/o scholars using Transana software 3.32 [46]. Coders identify with different levels of religious practices from different religious backgrounds and have advanced training working with Latina/o communities. Content analysis was utilized to answer our two research questions via a bottom–up approach [47]. Specifically, we conceptualized difficult migration experiences as stressors [48] and explored the role of religious practices as coping mechanisms during these stressors.

The analytic procedure began with a line-by-line bottom–up coding of the entire transcriptions to formulate initial categories, organize the data, and develop a codebook. The three coders met weekly to discuss the independent coding and any disagreements for each specific code. Coders also carried conceptual conversations around broad themes that emerged from distinct codes relating to the two research questions. For example, we observed how intrinsic aspects of religiosity were engaged during the migration process whereas extrinsic and social aspects of religiosity were explored during the U.S. lived experiences. Agreements and disagreements were always discussed in detail throughout weekly coding meetings until 100% agreement was reached. The final coding scheme was applied twice to the data to ensure that all relevant responses were coded accurately.

## 3. Results

### 3.1. Reasons for Immigration

Participants reported having entered the U.S. through a variety of ways including walking through the desert (30%), with a visa (5%), or as a toddler with a guardian (5%). A couple of the participants did not recall their migration experience due to early migration (10%) and a few others chose not to share how they entered the country (30%). Overall, findings revealed three primary reasons that motivated participants to come to the U.S. Nearly all participants (93%) identified existing family connections as the primary factor for migrating to the U.S. Many individuals described having parents or relatives living in the U.S. and were motivated to regain the family harmony and physical unity. Other participants described uniting with their husbands who were laborers in this country. The second most reported factor for migrating to the U.S. was for *“una mejor vida”* (64%), which translates to having a better life. Individuals reported living in poverty or having lower socio-economic status in their native countries, which motivated them to gain more financial resources through laboring in the U.S. By migrating, participants believed that they would attain better financial stability, better life satisfaction, and a higher quality of life. Lastly, the third most commonly reported factor for immigrating to the U.S. was conflict in their native country—therefore, loss of safety (20%). Some participants reported starting a new chapter of life in a new country after experiencing gun violence, domestic violence, and for some, community violence in their native country (e.g., Mexico). Overall, these themes support the CORT framework and suggest that reasons for migration are connected to efforts to preserve, shield, and nurture valued resources, and thus efforts for sustained stress reduction may have been present.

### 3.2. The Role That Religious Practices Play on the High-Stress Migration Process

Our content analysis provided important insights into how religious practices, primarily internal forms of religious practices such as prayers and reciting spiritual verses, played an important role during the migration process. We identified four distinct ways in which these religious practices sustained participants during the high-stress migration process: by providing strength, peace/serenity, reliance on a higher power, and gained/regained purpose during the migration experience. Each of these is discussed below.

**Provided Strength.** Many participants reported engaging in the religious practice of prayer, defined by them as direct communication with a higher power (e.g., saints and God). For these participants, especially those that migrated through means that produced excessive stress, such as crossing the desert (25%), prayer served as a source of emotional support during times of adversity. These participants relied on prayer as a source of stability that gave them physical and emotional strength. Juana, a 30-year-old, who identified as Protestant, described how prayer was used as a source of strength that provided solace at stressful times throughout her migration process. She noted the following:

*“It [prayer] strengthens me from my most difficult moments [walking through the desert], as I say it is where I take refuge and comfort. It is where it gave me the strength to continue [to walk through the desert].”*

For Juana and other Latinas/os, who migrated through stressful means, this form of religious practice was one that was accessible, feasible, and, most importantly, that provided potency and encouragement to continue through the process of crossing the border.

**Provided peace.** Other participants mentioned that they felt lonely during the migration process, especially when traveling alone. Some mentioned feeling nostalgic and missing people (like family) from their hometown immediately when the migration process (i.e., when they embarked on the journey to cross the border) began. Others reported that as soon as they departed their home communities, they became uncertain, often questioning the decisions made. These participants mentioned that when they felt alone, they found peace in communicating to a higher power. For many, this higher power was described as God or Mary. Luis, a 35-year-old, who identified as Catholic, described the following:

*“For the reason that I was away from my family, I asked God that they [family] were well, I asked him [God] to help me, because being alone is difficult. At the same time, I asked for my family back in Mexico, to see them again. That gave me peace.”*

Participants, like Luis, said that as soon as they began the migration process, they felt alone, nostalgic and often homesick, missing their family and close networks. They described that engaging in this religious practice brought serenity to their situation, often describing that their higher power had control of their situation.

**Provided reliance towards a higher power.** Other participants reported that one way to preserve and nurture psychological peace, physical endurance, and overall well-being when resources were lost throughout the migration process was by relying on a higher power. When further probed on how this is perceived as different than prayer, participants described that prayer is a means to communicate with a higher power (i.e., talking to God) and entrusting God, but they noted the difference between acting on a communicating mechanism or simply cognitively and emotionally entrusting their migration stress on a higher power. Several of these participants mentioned that reliance on a higher power was the first mechanism to prayer but this—at times—was the final outcome. Additionally, entrusting themselves to God helped them accept the trials faced when they were crossing the border. This was described as a sense of connection to something bigger than themselves when migrating to the U.S. Mario, a 28-year-old, who identified as Pentecostal, stated the following:

*“I have always trusted that if God brought me to America, it was for something. Whenever something good or bad happened [during the migration process], it was because of God. God knows why he did things. I therefore took the good side.”*

For Mario, his experience, while migrating to the U.S., was a difficult one. Although he perceived many lost resources while migrating, he saw it all as providential. He further described that trusting God allowed him to be more optimistic during the migration process and less stressed during challenging situations. Others mentioned that their reliance on a higher power protected them from dangers during the migration process. For instance, Juana stated that reliance on a higher power protected her during the physically-extenuating process of walking through the desert. She stated the following:

*“Well, I think [God] protected us a lot. [God] protected us a lot because during that walk [through the desert], we were finding everything wrong; it seems not, but [we] were finding dangers, and I thank God nothing happened to us.”*

**Gained/regained purpose.** Other participants described that engaging in religious practices allowed them to have a sense of purpose in their lives. Even in high-stress situations during their migration process, they at times felt there was a broader purpose by simply reverting to socialized religious messages (e.g., via spiritual readings, or messages heard in churches). For these participants, holding on to purpose-driven messages allowed them to have hope that difficulty and stress would subside. In the words of one of the participants, *“Our sense of purpose helps us think about what we can do.”* Other participants said that their sense of purpose came from higher powers and so they accepted their circumstances because everything happened for a reason. For many, this allowed them to focus on the overall reason that they migrated in the first place, which was to retain, preserve, and nurture valued resources in their native country. In doing so, the short-term stress experienced during their migration was minimized, given that their long-term sense of purpose would yield a greater outcome. For example, Jose, a 31-year-old who identified as Christian, stated the following:

*“Everywhere I was, I always tried to focus and look at God over my life and the necessities of our fellowmen… to be able to help as much as one can materially, spiritually, or emotionally.”*

Overall, common themes reveal that religious practices, primarily internal forms of religious practices, play an important role in helping individuals cope with high-stress situations (often due to lost resources) encountered during the migration process. These religious practices were frequently described as prayers, recitations of religious/spiritual content, communication with a higher power, and reliance toward a higher entity. Like the CORT framework, Latinas/os from our sample reported that engaging in these religious practices provided strength, peace/serenity, and helped them gain/regain purpose during the migration experience therefore mitigating stress in a high-stress situation.

### 3.3. The Role That Religious Practices Play on the U.S. Lived Experiences

Religious practices continued to shape the lived experiences of Latina/o immigrants in the U.S., where they encountered additional stressors (e.g., language barriers, financial stress, acculturative stress, and discrimination) during the adaptation process. Reviews of transcripts revealed the ways that religious practices helped them cope with the stressors associated with life in the U.S. Specifically, participants described that religious practices positively impacted their post-migration experiences in three main ways: by providing a community (e.g., church meetings and group activities), gained financial and social resources, and focused to do good. These are now discussed.

**Providing a community.** Several participants reported that engaging in religious practices (e.g., going to church) as newly arrived immigrants in the U.S. allowed them to find a community with similar religious and cultural values and beliefs. Many described missing their hometowns and families and that going to church allowed them to have a space that reminded them of their home country, where members of the congregations (e.g., church) spoke a similar language and also originated from a Latin American country. These findings suggest that being part of a congregation serves as a protective factor during stress-inducing situations because they did not feel alone. Rosa, a 30-year-old Protestant Christian, stated the following:

*“It’s [members from church] like a family, and it feels comfortable talking to the people that we knew before. We all see each other as a family, like a real brother and sister. And I have realized that there are no differences if a new one arrives. We greet her/him like if we already knew her/him. It is beautiful. It is beautiful to coexist in church with [our] brothers and sisters.”*

**Gained financial and social resources.** Many participants reported that engaging in religious practices, especially those that are public in nature (e.g., church attendance) assisted them in addressing various social needs. Several participants described facing many financial stressors upon arriving to the U.S., especially when they were not employed. Religious organizations, especially smaller churches, played an important role by providing them financial and material support, such as access to food banks, shelters, and social assistantships. Miguel, a 35-year-old who identified as Pentecostal, noted the following:

*“They [the church] knew that I was alone that I had not had a job at that time. And the pastor put together an offering and they gave it to me.”*

**Focused to do good.** Some participants expressed that they were exposed to more risks in the U.S. For instance, some reported that not having family could lead to the loss of cultural and protective values, such as the value for family. Other participants reported that there was more access to substances and drugs in the U.S. Similarly, others perceived that it was easier to experience mental health conditions (like depression and anxiety) in the U.S. (more than in their native country) due to exposure to additional stressors. Rafael, a 19-year-old, who identified as Catholic, reported the following:

*“If I had not been religious, with the easiness of living here [in America], like the ease of obtaining and consuming drugs and alcohol, it would have been very easy to fall into these risky behaviors. First, because of the loneliness and sadness. The money that one earns here [in America] also facilitates these risky behaviors - to take the wrong path.”*

Overall, religious practices continued to shape the lived experiences of most participants during the post-migration period, in which participants encountered additional stressors (e.g., language barriers, financial stress, acculturative stress, and discrimination). Churches provided participants not only with financial and social support, but also with a community of individuals that share similar values. Religious practices played a vital role in reducing stressors (especially perceived lost resources) for participants as they acclimated to a new country. Similar to what we found during the migration phase, religious practices served as a protective coping strategy during the post-migration phase when participants aspired to attain and preserve valued resources in the U.S., as described through the CORT framework.

## 4. Discussion

The goal of this qualitative study was to investigate the role of religious practices during the migration process and the U.S. lived experiences among recently arrived Latina/o immigrants. To this end, we conducted semi-structured interviews with 20 Latina/o immigrants living in a southern U.S. state. Our participants indicated that they migrated to the U.S. for three main reasons: to reunite with family members, for financial reasons given conditions of precarity, and/or due to a loss of safety in their native country (i.e., gun violence, domestic violence, and in some cases even community violence). In general, these findings are consistent with the CORT framework in that the decision to migrate is often marked by a loss of resources, which in this case is represented as the loss of social support after the migration of family members [48], loss of safety due to socio-political conflict [41], and/or financial stress [4,28]. These findings suggest that these lost resources may be a driving force to migrate to a new country to restore the family unit, acquire safety, and reduce financial stress.

Results also demonstrated that during the migration process, religious practices, primarily internal forms of religious behaviors (e.g., prayers, reciting spiritual verses, entrusting themselves to God), played an important role in coping with stressors. This finding is supported by the overarching literature on forms of religious coping among Latina/o communities, especially those with limited resources during high-stress state ([49]). The findings presented here expand the literature by further elucidating the central role that these religious practices have in providing strength, peace/serenity, and gaining/regaining purpose during the migration process. Future studies should examine the role of religious practices and behaviors in the context of different migration experiences (e.g., undocumented vs. legal migration).

Findings also reveal that upon arriving in their host country, religious practices continued to shape their U.S. lived experiences of immigrants as they encounter additional stressors associated with language barriers, financial stress, acculturative stress, discrimination, etc. This finding corroborates the overarching literature that supports the lived experiences of oppressed groups, especially recently arrived Latina/o immigrants [2,12]. Our data contributes to this literature by revealing how these religious practices (primarily practices that include social means like church attendance) shaped participants’ lived experiences in the U.S., specifically by providing a community, assisting in financial and social support, and focused to do good. This novel finding supports the CORT framework given that this community may encounter additional losses upon arriving to the U.S. [27,44]. For example, Latina/o immigrants encounter financial and work-related stress [4,11], acculturative stress [12], and various forms of discrimination [2], yet have limited access to mental health care [7,8,9,10]. The CORT framework supports the theory that these religious practices play a key role in stress reduction during their high-stress experiences. Our findings suggest that prayer, church attendance, and other religious behaviors serve a crucial protective role for Latina/o immigrants in the context of high stress and low access and utilization of mental care services. One key point to emphasize is that participants in fact referred mainly to personal prayer and religious communities to which they belonged to once they arrived to the U.S. This religious experience may be connected specific to this population and their culturally specific needs; however, these religious experiences may evolve as they spend more time in the U.S. Research can continue to examine other domains of religious experiences among Latina/o immigrants after several years in the U.S., and especially as they begin to acculturate.

Given that religious practices play a central role in the lives of Latina/o migrants [37], interdisciplinary scholars and practitioners working with the Latinx and migrant communities would benefit from understanding the lived experiences of these communities via a CORT framework. That is, scholars and practitioners can further understand what Latina/o migrants are aiming to attain, preserve, shield, and nurture. This would assist in conceptualizing different stress levels across individuals in the same communities. Additionally, interdisciplinary scholars and practitioners can further collaborate with faith-based organizations in efforts to provide supportive means to reduce perceived high-stress situations shortly after migration. Most importantly, interdisciplinary scholars and practitioners can translate science into practice by implementing programs that provide better care for this population in culturally relevant means. For example, our findings suggest specific types of religious practices (e.g., forms of prayer, communal or personal nature of practices). Having a thorough understanding of what religious experience means for these communities can play a critical role in disseminating and implementing culturally sensitive programs for these communities. Furthermore, by working with faith-based organizations through a community-based approach, interdisciplinary scholars and practitioners can build rapport with the community and use their leaders to disperse information about physical and behavioral health programs. Finally, engaging in these collaborative approaches can also help address the underutilization of overall health services among the Latina/o communities.

## 5. Limitations and Strengths

There are several limitations that are worth noting. While our study sheds light on the role of religious practices in the lives of Mexican immigrants, findings may not be generalizable to immigrants from other Latin American countries. Future research should examine whether similar or different patterns are observed among other immigrant groups. Second, due to its sensitive nature, we did not ask participants about their legal status nor length of residence in the U.S. Still, participants volunteered some information by describing experiences of entering the U.S. through alternative legal crossing (e.g., crossing through the river or a desert). Future research should further explore the role of religious practices on the lived experiences of undocumented vs. documented migrants. Despite the limitations mentioned above, this study has several strengths. First, the qualitative methodology allowed us to learn how immigrants interpret their own experiences and the role of religious practices and behaviors in their lives. In addition, although there is a growing body of literature that examines the relationship between religiosity among Latinas/os in the U.S., this study provides additional insight to Latina/o immigrants’ experiences and the protective role of religious practices.

## 6. Conclusions

Findings from this study provide great insight into the role that religious practices play on the migration process and the U.S. lived experiences of Latina/o immigrants. The different findings revealed that internal forms of religious behaviors were primarily seen during the migration process and external forms of religious behaviors were primarily seen upon arriving to the U.S. These findings highlight how religious behaviors are complex and manifest differently depending on the stage of the migration experience. Given that more than 80% of Latinas/os immigrants identify as religious [13], clinicians who work with recently arrived Latina/o immigrants should be knowledgeable about, and sensitive to, the context associated with the role of religious practices during the pre- and post-migration process and lived experiences.
